# LINC00665 promotes Ovarian Cancer progression through regulating the miRNA-34a-5p/E2F3 axis

**DOI:** 10.7150/jca.51457

**Published:** 2021-01-18

**Authors:** Dan Xu, Qingxia Song, Ying Liu, Wansu Chen, Lijuan LU, Min Xu, Xiaohui Fang, Wenjie Zhao, Huifang Zhou

**Affiliations:** 1 Department of Gynaecology, Suzhou TCM Hospital Affiliated to Nanjing University of Chinese Medicine, Suzhou 215009, China.; 2 Department of Gynaecology, Affiliated Hospital of Nanjing University of Chinese Medicine, Nanjing 210029, China.; 3 Nanjing University of Chinese Medicine, Nanjing, 210023, China.; 4 Department of clinical laboratory, Suzhou TCM Hospital Affiliated to Nanjing University of Chinese Medicine, Suzhou 215009, China.

**Keywords:** ovarian cancer, LINC00665, miRNA-34a-5p, E2F3

## Abstract

**Objective:** To clarify the role of LINC00665 in ovarian cancer (OC) progression and the possible mechanism.

**Methods:** LINC00665 levels in OC tissues and cell lines were detected by qRT-PCR. The correlation between LINC00665 and clinicopathologic characteristics of OC patients was assessed. Biological functions of OC cell phenotypes influenced by LINC00665 were examined by CCK-8, colony formation and Transwell assay. Dual-luciferase reporter assay and RIP assay were conducted to verify the interaction between LINC00665 and its downstream target.

**Results:** LINC00665 was upregulated in OC and linked to poor prognosis. Knockdown of LINC00665 blocked malignant proliferative, migratory and invasive functions of OC cells. By competitively binding miRNA-34a-5p, LINC00665 abolished the inhibitory effect of miR-34a-3p on its downstream gene E2F3, thus promoting OC progression.

**Conclusion:** LINC00665/miRNA-34a-5p/E2F3 axis is involved in OC progression, providing novel insights into the clinical treatment of OC.

## Introduction

Ovarian cancer (OC) is a reproductive system tumor that seriously endangers women's lives, and its mortality ranks first among gynecological malignancies 1.

Because of its insidious symptoms, only 20% of OC cases can be detected in early examinations 2. Many patients have abdominal metastases before the onset of symptoms. Seriously, most OC patients are in the advanced stage when they are diagnosed for the first time. The 5-year survival of advanced OC is only 10-20% 3, 4. Epithelial ovarian cancer (EOC) accounts for about 90% of all ovarian malignancies. In recent years, surgery combined with chemotherapy and the latest neoadjuvant chemotherapy has elevated therapeutic efficacy of OC, its recurrence rate remains high 5, 6. Therefore, it is urgent to study the underlying mechanism of the malignant progression of OC. Seeking for biomarkers of OC helps to guide clinical treatment. LncRNAs are noncoding RNAs longer than 200 nucleotides in transcripts. A growing number of studies have shown that lncRNAs can interact with RNAs, DNAs and proteins, thereby regulating gene transcription and post-transcription 7, 8. LncRNAs have been detected to be abnormally expressed in malignant tumors, serving either as oncogenes or tumor-suppressor genes 9, 10. Interestingly, the number of different lncRNAs associated with OC, and consequently publications on the topic, has recently grown exponentially. Increasing studies have indicated that abnormal expressions of lncRNAs play a crucial role in the occurrence progression and treatment of ovarian cancer 11, 12. Pan et al. 13 reported that LINC00339 was upregulated in ovarian cancer tissues and cell lines, and promoted cell proliferation, migration, and invasion of ovarian cancer via regulation of miR-148a-3p/ROCK1 axes. Zhang et al. 14 reported that the overexpression of lncRNA, MLK7-AS1, promoted ovarian cancer cell progression by modulating the miR-375/YAP1 axis. Yang et al. 15 reported that miR-200c overexpression inhibits the invasion and tumorigenicity of epithelial ovarian cancer cells by suppressing lncRNA HOTAIR in mice, suggesting that the miR-200c and lncRNA HOTAIR could be effective therapeutic targets for human epithelial ovarian cancer treatment. LINC00665 is located on chromosome 19. It is upregulated in multiple types of tumors as an oncogene. It is reported that LINC00665 is upregulated in lung carcinoma cases and correlated to a poor prognosis. LINC00665 triggers lung carcinoma progression by sponging miR-98 and thus mediates the ERK signaling 16. In addition, LINC00665 is also involved in the progression of gastric cancer and breast cancer 17-19. Its biological function in OC is largely unclear.

Using TCGA database and clinical tissues collected in our center, we first examined LINC00665 level in OC and its clinical significance. Later, its regulatory effects on OC cell functions* in vitro* were detected. Our findings revealed the important role of the LINC00665/miRNA-34a-5p/E2F3 axis in OC progression, providing novel targets for the treatment of OC.

## Methods

### Clinical samples

Surgically resected EOC (n=56) and non-tumor tissues (n=56) were pathologically detected and stored in liquid nitrogen. Patients did not have preoperative adjuvant therapy. We recorded clinical data of them for analysis (Table 1). Collection of samples for experimental use was informed consent and approved by the Ethic Committee of Suzhou TCM Hospital Affiliated to Nanjing University of Chinese Medicine.

### Cell culture and transfection

OC cell lines (A2780, OVCAR3, CAOV3 and SKOV3) and ovarian epithelial cell line (IOSE80) were purchased from Cell Bank, Chinese Academy of Sciences, Shanghai, China. Cells were cultivated in RPMI-1640 containing 10% FBS (Gibco, USA) at 37 °C, 5% CO_2_.

Three lines of LINC00665 shRNAs and the negative control were produced by GenePharma, Shanghai, China. MiRNA-34a-5p mimics, inhibitor and negative control were provided by RiboBiotech, Guangzhou, China. Using Lipofectamine 2000 (Invitrogen, USA), plasmids were transfected into cells.

### qRT-PCR

Tissue or cell samples were processed by TRIzol for isolating RNAs. After purification, qualified RNAs were reversely transcribed to cDNAs and subjected to qRT-PCR using SYBR®Premix Ex Taq™ (Takara, Japan). Relative levels of PCR products were calculated by 2^-ΔΔCt^ and normalized to that of GAPDH using the StepOne Plus Real-Time PCR system (ABI, USA). Primer sequences were listed as follows.

- LINC00665: Forward: 5ʹ-GGTGCAAAGTGGGAAGTGTG-3ʹ;
- Reverse: 5ʹ-CGGTGGACGGATGAGAAACG-3ʹ;
- miRNA-34a-5p: Forward: 5ʹ-CCCAGAACATAGACACGCTGGA-3ʹ;
- Reverse: 5ʹ-ATCAGCTGGGCACCTAGGACA-3ʹ;
- E2F3: Forward: 5ʹ-AGAAAGCGGTCATCAGTACCT-3ʹ;
- Reverse: 5ʹ-TGGACTTCGTAGTGCAGCTCT-3ʹ;
- U6: Forward: 5ʹ-CTCGCTTCGGCAGCAGCACATATA-3ʹ;
- Reverse: 5ʹ-AAATATGGAACGCTTCACGA-3ʹ;
- GAPDH: Forward: 5ʹ- GGAGCGAGATCCCTCCAAAAT-3ʹ;
- Reverse: 5ʹ- GGCTGTTGTCATACTTCTCATGG -3ʹ.

### Western blot

Total protein was extracted using RIPA containing trypsin (Roche, USA), following 20-min centrifugation at 12,000 rpm. Protein concentration was detected using the BCA method (Pierce, USA). In each lane, 50 μg protein sample was separated by SDS-PAGE and loaded on PVDF membranes (Millipore, USA), which were incubated in 5% skim milk for 2 h. Membranes were immunoblotted with primary (anti-E2F3, GAPDH) and secondary antibodies (anti-mouse, anti-rabbit; Cell Signaling Technology). Band exposure was achieved by ECL.

### CCK-8 assay

Cells were seeded in a 96-well plate with 2×10^3^ cells suspended in 200 μL of medium per well. They were induced with 10 μl of CCK-8 solution per well at the indicated time points. After cell culture in the dark for 2 h, absorbance at 490 nm was detected for plotting cell viability curves.

### Colony formation assay

Cells in a 6-well plate with 1×10^3^ cells/well were cultured for 10 days. Colonies were then fixed, dyed and captured for counting.

### Transwell assay

Serum-free suspension (2×10^5^ cells /ml) was seeded in a Transwell chamber either pre-coated with Matrigel or not. Serum-contained medium was added to the bottom as an inducer. After 24-h cell culture, cells penerated to the bottom were fixed, and dyed in 0.2% crystal violet. They were captured in 5 random fields per well for cell counting.

### Dual-luciferase reporter assay

Luciferase vectors LINC00665-WT, LINC00665-MUT, E2F3-WT and E2F3-MUT were purchased from RiboBiotech (Guangzhou, China). SKOV3 cells seeded in a 24-well plate were co-transfected with luciferase vectors and miRNA-34a-5p mimics/miR-NC for 48 h, followed by measurement of luciferase activity using the dual-luciferase reporter assay system (Promega, USA).

### RIP assay

RIP assay was conducted based on protocols of the Magna RIP RNA-Binding Protein Immunoprecipitation Kit (Millipore, USA).

### Statistical analysis

SPSS 22.0 was used for statistical analysis. Differences between groups were compared by the *t*-test. Kaplan-Meier method was introduced for survival analysis, followed by log-rank test. Correlation between expression levels of two genes was assessed by Pearson correlation test. *P* < 0.05 was considered as statistically significant.

## Results

### LINC00665 was upregulated in OC and correlated with poor prognosis

**Figure 1:**
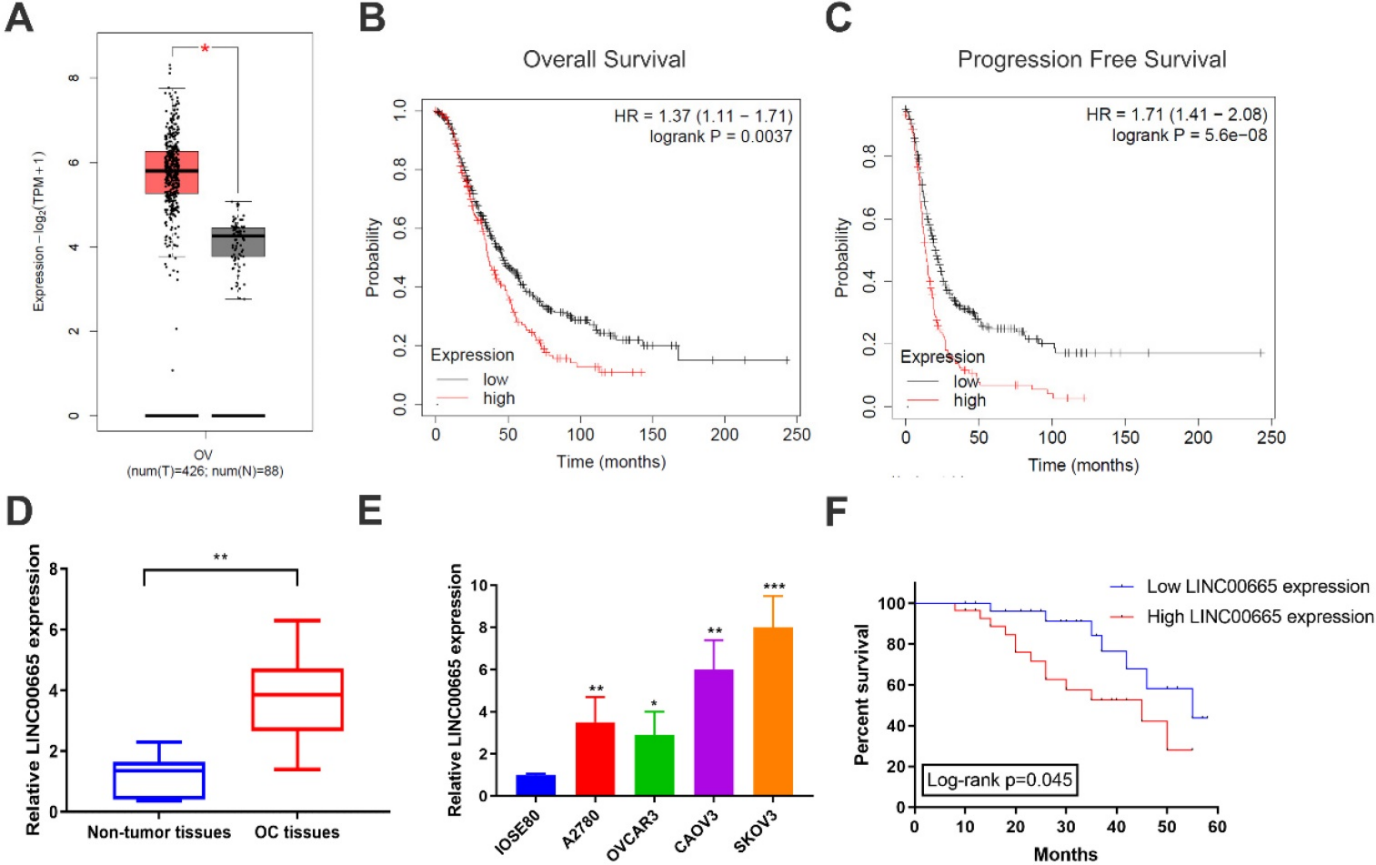
** LINC00665 was upregulated in ovarian cancer.** (A) LINC00665 expression was significantly increased in ovarian cancer tissues compared to normal tissues in TCGA database. (B,C) High LINC00665 expression was significantly associated with poor overall survival (B) and progression free survival rate (C) of ovarian cancer patients in Kaplan-Meier Plotter database. (D) Expression of LINC00665 in ovarian cancer and adjacent non-tumor tissues was measured by qRT-PCR. (E) Expression of LINC00665 in ovarian cancer cell lines was measured by qRT-PCR. (F) Kaplan-Meier curve was plotted to analyze patients' overall survival rate based on LINC00665 expression. (* p<0.05, ** p<0.01, *** p<0.001)

Using TCGA database, it is found that LINC00665 was upregulated in OC tissues than controls (Figure 1A), which was validated by detecting LINC00665 level in clinical samples of OC in our center (Figure 1D). Compared with ovarian epithelial cells, LINC00665 was highly expressed in OC cell lines (Figure 1E). Subsequently, as data revealed in Kaplan-Meier Plotter, LINC00665 was correlated to overall survival and progression-free survival of OC (Figure 1B, C). To further analyze the clinical significance of LINC00665 in OC, recruited patients were divided to two groups based on the median level of LINC00665 in corresponding cancer tissues. Survival analysis showed that high level of LINC00665 was unfavorable to the survival of OC (Figure 1F). Moreover, LINC00665 level was correlated to tumor size, FIGO stage and lymph node metastasis, rather than the age, histological grade, pathological subtype and CA125 level (Table 1). It is concluded that LINC00665 was upregulated in OC and correlated to the prognosis.

### Knockdown of LINC00665 inhibits proliferative and metastatic capacities of ovarian cancer cells

**Figure 2:**
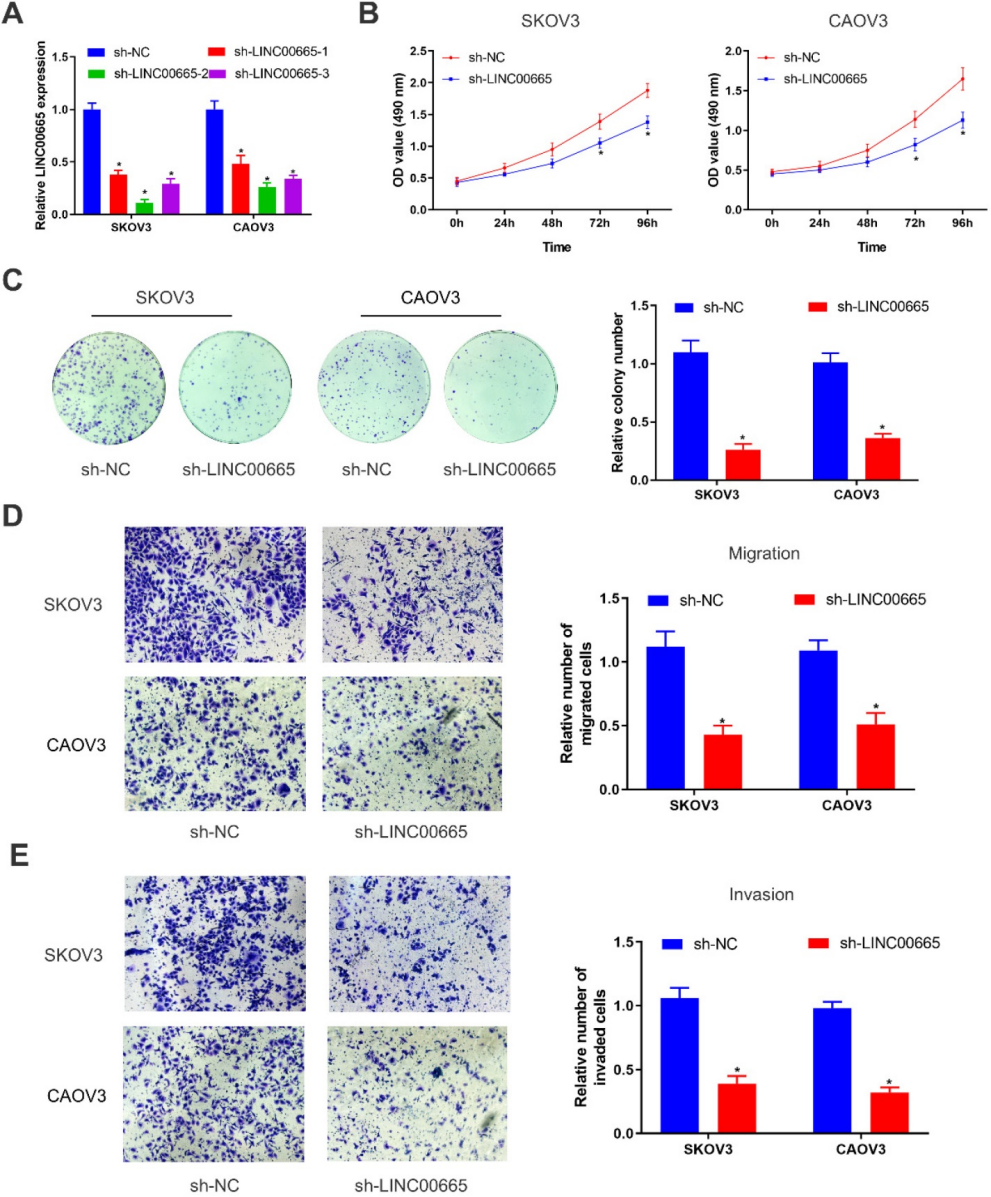
** Depletion of LINC00665 inhibits ovarian cancer cell proliferation, migration, and invasion.** (A) Expression of LINC00665 in SKOV3 and CAOV3 cells transfected with sh-NC or sh-LINC00665 was determined by qRT-PCR. (B,C) Cell growth was evaluated by CCK8 assay (B) and colony formation assay(C) in the SKOV3 and CAOV3 cells transfected with sh-NC or sh-LINC00665. (D,E) Cell migration (D) and invasion (E) was measured by Transwell assay in SKOV3 and CAOV3 cells transfected with sh-NC or sh-LINC00665. (* p<0.05)

We constructed LINC00665 knockdown model in SKOV3 and CAOV3 cells by lentivirus transfection, and its intervention efficacy was examined by qRT-PCR (Figure 2A). Among the three lines of sh-LINC00665, sh-LINC00665-2 displayed the highest efficacy, and it was used in the following experiments. CCK-8 and colony formation assay obtained the finding that knockdown of LINC00665 reduced proliferative rate in SKOV3 and CAOV3 cells (Figure 2B, C). Besides, migratory and invasive rates of OC cells were declined after transfection of sh-LINC00665-2 (Figure 2D, E).

### LINC00665 binds to miRNA-34a-5p and negatively regulates its level

**Figure 3:**
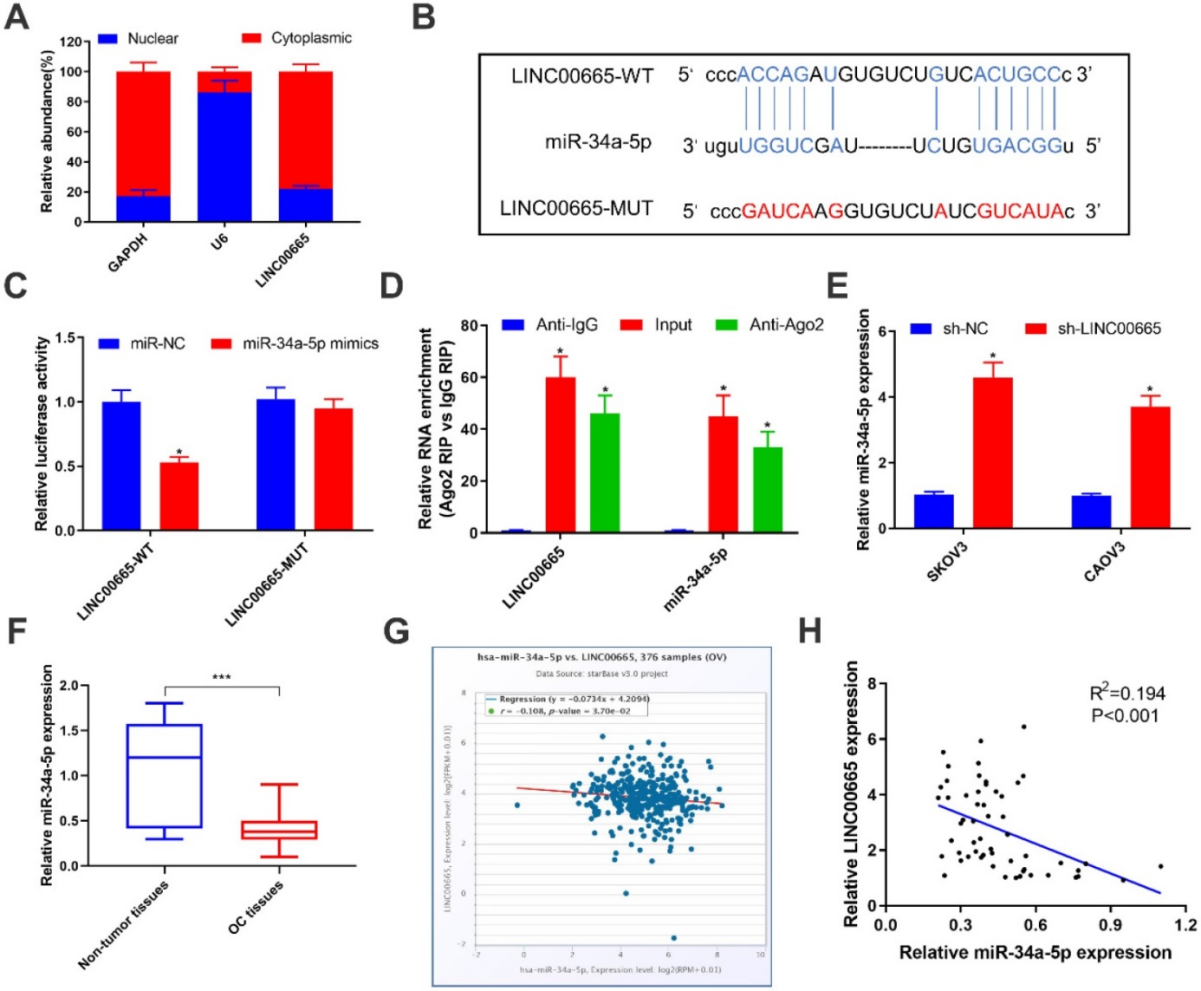
** LINC00665 binds to miR-34a-5p and represses its expression.** (A) Relative LINC00665 expression levels in the nuclear and cytoplasmic fractions of SKOV3 cells. (B) The putative binding sequence sites in LINC00665 with miR-34a-5p. The red color-highlighted sequence was the mutated site in LINC00665. (C) Dual luciferase reporter assay was performed to validate the interaction between miR-34a-5p and LINC00665 in SKOV3 cells. (D) RIP analysis of endogenous AGO2 binding to RNA in SKOV3 cells. IgG was used as the control. Expression of LINC00665 and miR-34a-5p was determined by qRT-PCR. (E) Expression of miR-34a-5p in SKOV3 and CAOV3 cells transfected with sh-NC or sh-LINC00665 was determined by qRT-PCR. (F) Expression of miR-34a-5p in ovarian cancer and adjacent non-tumor tissues was measured by qRT-PCR. (G,H) Negative correlation was observed between LINC00665 and miR-34a-5p in ovarian cancer tissues according to TCGA database (G) and our center data (H). (* p<0.05, ** p<0.01, *** p<0.001)

To further explore the oncogenic mechanism of LINC00665, subcellular distribution of LINC00665 was identified. It is shown that LINC00665 was mainly expressed in the cytoplasm (Figure 3A). Cytoplasmic lncRNAs can competitively bind miRNAs and thus exert the miRNA sponge effect 20. Bioinformatic prediction depicted a binding sequence in miRNA-34a-5p 3'UTR pairing to that of LINC00665 (Figure 3B). Later, we constructed LINC00665-WT and LINC00665-MUT vectors for dual-luciferase reporter assay. Overexpression of miRNA-34a-5p markedly decreased luciferase activity in cells transfected with LINC00665-WT, rather than those transfected with LINC00665-MUT (Figure 3C). In addition, LINC00665 and miRNA-34a-5p were mainly enriched in anti-Ago2, suggesting the interaction between LINC00665/miRNA-34a-5p and Ago2 (Figure 3E). MiRNA-34a-5p was markedly downregulated in OC tissues (Figure 3F, G). In OC cells transfected with sh-LINC00665-2, miRNA-34a-5p was upregulated (Figure 3E). As expected, we obtained a negative correlation between LINC00665 and miRNA-34a-5p in OC tissues (Figure 3H). Taken together, LINC00665 regulated OC progression by targeting miRNA-34a-5p as a ceRNA.

### LINC00665 regulated E2F3 by competitively binding miRNA-34a-5p

**Figure 4:**
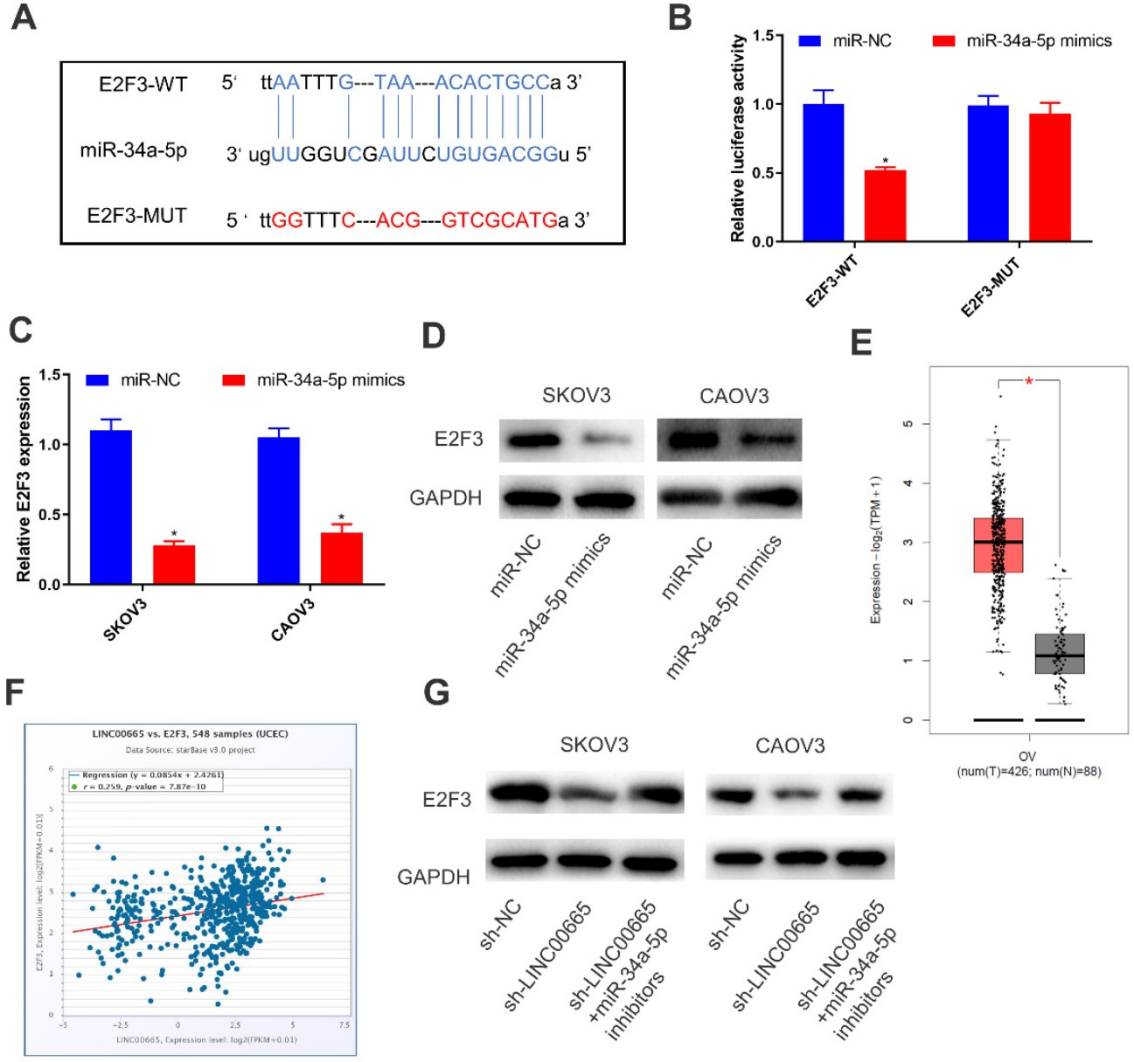
** LINC00665 regulated E2F3 by absorbing miR-34a-5p.** (A) The putative binding sequence sites in E2F3 with miR-34a-5p. The red color-highlighted sequence was the mutated site in E2F3. (B) Dual luciferase reporter assay was performed to validate the interaction between miR-34a-5p and E2F3 in SKOV3 cells. (C, D) Expression of E2F3 in SKOV3 and CAOV3 cells transfected with miR-NC or miR-34a-5p mimics was determined by qRT-PCR (C) and Western Blot (D). (E) E2F3 was highly expressed in ovarian cancer tissues compared to normal tissues in TCGA database. (F) Positive correlation was observed between LINC00665 and E2F3 in ovarian cancer tissues according to TCGA database. (G) E2F3 protein expression in SKOV3 and CAOV3 cells transfected with sh-NC, sh-LINC00665 or sh-LINC00665+ miR-34a-5p inhibitors was determined by Western Blot. (* p<0.05)

By comprehensively analyzing online databases (TargetScan, miRDB and Starbase), E2F3 was predicted to be the downstream gene of miRNA-34a-5p, which was further verified by dual-luciferase reporter assay (Figure 4A, B). Both mRNA and protein levels of E2F3 were downregulated in SKOV3 and CAOV3 cells overexpressing miRNA-34a-5p (Figure 4C, D). To further clarify the interaction in the LINC00665/miRNA-34a-5p/E2F3 axis, we analyzed E2F3 level in OC tissues in TCGA, and the results showed that E2F3 was upregulated (Figure 4E). Moreover, E2F3 level was positively correlated with that of LINC00665 (Figure 4F). Protein level of E2F3 was downregulated by knockdown of LINC00665, while it was reversed by knockdown of miRNA-34a-5p (Figure 4G). Hence, LINC00665 upregulated E2F3 by sponging miRNA-34a-5p.

### LINC00665 promotes ovarian cancer progression by regulating the miRNA-34a-5p/E2F3 axis

**Figure 5:**
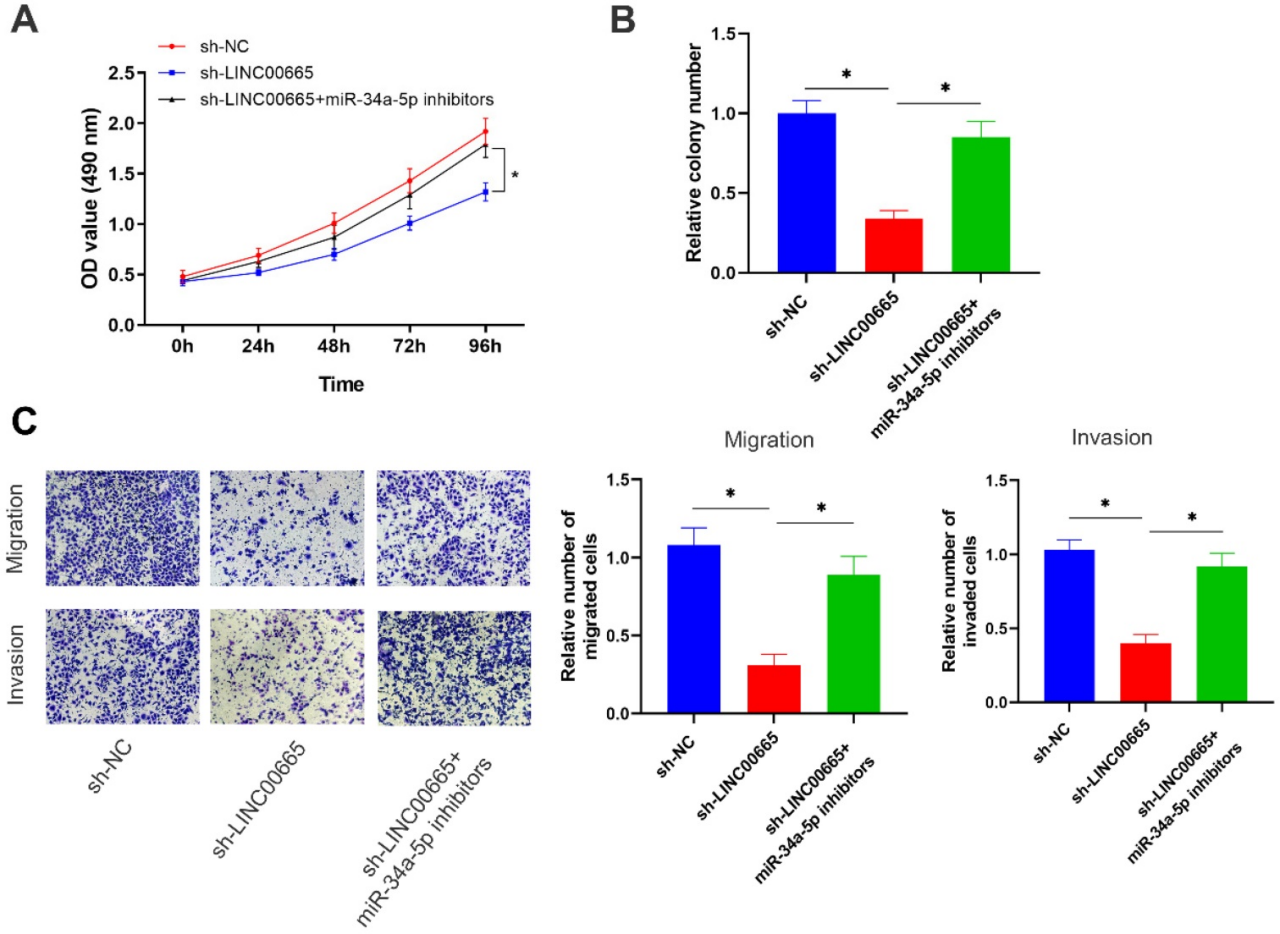
** LINC00665 promotes ovarian cancer progression by regulating the miR-34a-5p/E2F3 axis.** (A, B) Cell proliferation was measured by CCK8 assay (A) and colony formation assay (B) in the SKOV3 cells transfected with sh-NC, sh-LINC00665 or sh-LINC00665+ miR-34a-5p inhibitors. (C) Cell migration and invasion was measured by Transwell assay in SKOV3 cells transfected with transfected with sh-NC, sh-LINC00665 or sh-LINC00665+ miR-34a-5p inhibitors. (* p<0.05)

Rescue experiments were conducted to assess whether the miRNA-34a-5p/E2F3 axis was involved in the regulation of OC progression by LINC00665. Reduced proliferative rate of OC cells with LINC00665 knockdown was reversed by intervention of miRNA-34a-5p (Figure 5A, B). In addition, migratory and invasive rates were higher in OC cells with co-knockdown of LINC00665 and miRNA-34a-5p than those with single knockdown of LINC00665 (Figure 5C). Knockdown of miRNA-34a-5p was able to reverse the role of LINC00665 in regulating OC cell functions.

## Discussion

With the development of high-throughput sequencing, dysfunctional lncRNAs are believed as key events for tumor development 21. Recent studies have shown that LINC00665 exerts a carcinogenic role in many types of tumors 17-19.

Our results revealed that LINC00665 was upregulated in OC tissues, which was validated in online analysis using TCGA database. Clinical data of recruited OC patients were retrospectively reviewed and it is shown that LINC00665 was correlated to tumor size, FIGO stage and lymph node metastasis of OC. High level of LINC00665 predicted a poor survival of OC patients. *In vitro* evidences demonstrated that knockdown of LINC00665 weakened proliferative, migratory and invasive capacities of OC cells.

LncRNAs can regulate gene transcription, post-transcription and translation depending on their intracellular location 22. MALAT1 is mainly located in the paraspeckle of cell nuclei, where RNA is spliced and modified, suggesting that MALAT1 is able to mediate alternative splicing of precursor mRNAs 23. LINC00665 was mostly expressed in the cytoplasm. We therefore speculated that LINC00665 could be a modifier for post-transcriptional regulation. Through competitively binding target miRNAs and therefore influencing the binding between miRNAs and the downstream genes, lncRNAs display their biological functions, that is, the ceRNA theory 24. Ji et al. 25 reported that LINC00665 drives breast cancer progression by sponging miR-379-5p and upregulating LIN28B. In the present study, miRNA-34a-5p was confirmed to be sponged by LINC00665 in OC cells, and a negative correlation between their levels was identified.

MiRNAs are promising diagnostic and prognostic biomarkers of OC 26. Previous studies have shown that miRNA-34a-5p is downregulated in multiple types of tumors 27, 28. MiRNA-34a-5p is capable of inhibiting proliferative function and reducing cisplatin resistance in OC cells 29. Here, E2F3 was proven to be the downstream gene of miRNA-34a-5p, which is a member of the E2F family displaying an oncogenic role 30. E2F3 level was positively correlated to LINC00665 level in OC tissues. As a result, the LINC00665/miRNA-34a-5p/E2F3 axis was ascertained. Rescue experiments obtained the results that miRNA-34a-5p could reverse the regulatory effects of LINC00665 on OC cell functions. Collectively, LINC00665 promoted OC progression by the miRNA-34a-5p/E2F3 axis.

## Conclusion

LINC00665 is upregulated in OC cases and predicts a poor prognosis. LINC00665/miRNA-34a-5p/E2F3 axis is involved in OC progression, providing novel insights into the clinical treatment of OC.

## Figures and Tables

**Table 1:** Association of LINC00665 expression with clinicopathologic characteristics of ovarian cancer

Parameters	Number of cases	LINC00665 expression		*p*-value
		Low (n=28)	High (n=28)	
**Age (years)**				0.420
<50	25	11	14	
≥50	31	17	14	
**Tumor size (cm)**				0.016*
<10 cm	29	19	10	
≥10 cm	27	9	18	
**FIGO stage**				0.035*
I-II	15	11	4	
III-IV	41	17	24	
**Histological grade**				0.422
G1-G2	29	13	16	
G3	27	15	12	
**Lymph node metastasis**				0.032*
No	30	19	11	
Yes	26	9	17	
**Histological subtype**				0.217
Serous	42	23	19	
Others	14	5	9	
**CA125 Level (U/ml)**				0.284
<500	26	15	11	
≥500	30	13	17	

*p<0.05.

## References

[1] Siegel RL, Miller KD, Jemal A (2020). Cancer statistics, 2020. CA Cancer J Clin.

[2] Boussios S, Mikropoulos C, Samartzis E (2020). Wise Management of Ovarian Cancer: On the Cutting Edge. J Pers Med.

[3] Yousefi M, Dehghani S, Nosrati R (2020). Current insights into the metastasis of epithelial ovarian cancer - hopes and hurdles. Cell Oncol (Dordr).

[4] Bookman MA (2019). Can we predict who lives long with ovarian cancer?. Cancer.

[5] Xie H, Wang W, Xia B (2020). Therapeutic applications of PARP inhibitors in ovarian cancer. Biomed Pharmacother.

[6] Koole SN, van Driel WJ, Sonke GS (2019). Hyperthermic intraperitoneal chemotherapy for ovarian cancer: The heat is on. Cancer.

[7] Zhao Y, Teng H, Yao F (2020). Challenges and Strategies in Ascribing Functions to Long Noncoding RNAs. Cancers (Basel).

[8] Fatica A, Bozzoni I (2014). Long non-coding RNAs: new players in cell differentiation and development. Nat Rev Genet.

[9] Peng Y, Tang D, Zhao M (2020). Long non-coding RNA: A recently accentuated molecule in chemoresistance in cancer. Cancer Metastasis Rev.

[10] Hu Q, Egranov SD, Lin C (2020). Long noncoding RNA loss in immune suppression in cancer. Pharmacol Ther.

[11] Martín SM, Mónica LM, Aida BA (2020). The Challenges and Opportunities of LncRNAs in Ovarian Cancer Research and Clinical Use. Cancers (Basel).

[12] Abildgaard C, Do Canto LM, Steffensen KD (2020). Long Non-coding RNAs Involved in Resistance to Chemotherapy in Ovarian Cancer. Front Oncol.

[13] Pan L, Meng Q, Li H (2019). LINC00339 promotes cell proliferation, migration, and invasion of ovarian cancer cells via miR-148a-3p/ROCK1 axes. Biomed Pharmacother.

[14] Yan H, Li H, Li P (2018). Long noncoding RNA MLK7-AS1 promotes ovarian cancer cells progression by modulating miR-375/YAP1 axis. J Exp Clin Cancer Res.

[15] Yang C, Li H, Zhang T (2020). miR-200c overexpression inhibits the invasion and tumorigenicity of epithelial ovarian cancer cells by suppressing lncRNA HOTAIR in mice. J Cell Biochem.

[16] Cong Z, Diao Y, Xu Y (2019). Long non-coding RNA linc00665 promotes lung adenocarcinoma progression and functions as ceRNA to regulate AKR1B10-ERK signaling by sponging miR-98. Cell Death Dis.

[17] Ding J, Zhao J, Huan L (2020). Inflammation-Induced Long Intergenic Noncoding RNA (LINC00665) Increases Malignancy Through Activating the Double-Stranded RNA-Activated Protein Kinase/Nuclear Factor Kappa B Pathway in Hepatocellular Carcinoma. Hepatology.

[18] Guo B, Wu S, Zhu X (2020). Micropeptide CIP2A-BP encoded by LINC00665 inhibits triple-negative breast cancer progression. EMBO J.

[19] Yang B, Bai Q, Chen H (2020). LINC00665 induces gastric cancer progression through activating Wnt signaling pathway. J Cell Biochem.

[20] Chan JJ, Tay Y (2018). Noncoding RNA: RNA Regulatory Networks in Cancer. Int J Mol Sci.

[21] Huarte M (2015). The emerging role of lncRNAs in cancer. Nat Med.

[22] Zhang XZ, Liu H, Chen SR (2020). Mechanisms of Long Non-Coding RNAs in Cancers and Their Dynamic Regulations. Cancers (Basel).

[23] Hochberg-Laufer H, Neufeld N, Brody Y (2019). Availability of splicing factors in the nucleoplasm can regulate the release of mRNA from the gene after transcription. PLoS Genet.

[24] Abdollahzadeh R, Daraei A, Mansoori Y (2019). Competing endogenous RNA (ceRNA) cross talk and language in ceRNA regulatory networks: A new look at hallmarks of breast cancer. J Cell Physiol.

[25] Ji W, Diao YL, Qiu YR (2020). LINC00665 promotes breast cancer progression through regulation of the miR-379-5p/LIN28B axis. Cell Death Dis.

[26] Staicu CE, Predescu DV, Rusu CM (2020). Role of microRNAs as Clinical Cancer Biomarkers for Ovarian Cancer: A Short Overview. Cells.

[27] Zheng F, Li J, Ma C (2020). Novel regulation of miR-34a-5p and HOTAIR by the combination of berberine and gefitinib leading to inhibition of EMT in human lung cancer. J Cell Mol Med.

[28] Haghi M, Taha MF, Javeri A (2019). Suppressive effect of exogenous miR-16 and miR-34a on tumorigenesis of breast cancer cells. J Cell Biochem.

[29] Zuo Y, Zheng W, Liu J (2020). MiR-34a-5p/PD-L1 axis regulates cisplatin chemoresistance of ovarian cancer cells. Neoplasma.

[30] Bellmunt J (2018). Stem-Like Signature Predicting Disease Progression in Early Stage Bladder Cancer. The Role of E2F3 and SOX4. Biomedicines.

